# The Key Target and Molecular Mechanism of the Volatile Component of *Scutellaria baicalensis* Georgi in Acute Lung Injury Based on Network Pharmacology

**DOI:** 10.3389/fphar.2021.650780

**Published:** 2021-04-26

**Authors:** Guosong Zhu, Jiaqiang Zhang, Yali Yang, Haoran Zhang, Wenwen Jin, Fangchu Su, Junting Liang, Kaiwei Wang, Jianhua Zhang, Chuanliang Chen

**Affiliations:** ^1^ Department of Anesthesia and Perioperative Medicine, Henan Provincial People's Hospital, People's Hospital of Zhengzhou University, Zhengzhou, China; ^2^ Medical Engineering Technology and Data Mining Institute, Zhengzhou University, Zhengzhou, China; ^3^ Clinical Bioinformatics Experimental Center, Henan Provincial People's Hospital, People's Hospital of Zhengzhou University, Zhengzhou, China

**Keywords:** key target, signal pathway, acute lung injury, network pharmacology, wogonin

## Abstract

**Ethnopharmacological relevance:** *Scutellaria baicalensis* georgi is one of the most widely studied TCMs; its effects in ALI have been studied in a large number of experiments, and the efficacy of volatile oil from TCM remains to be studied.

**Aim:** The volatile component of *Scutellaria baicalensis* georgi was selected to act on the key target of acute lung injury and was preliminarily studied for its specific molecular mechanism.

**Methods:** The volatile active substances of *Scutellaria baicalensis* georgi were extracted by GC–MS, and the active ingredients related with the occurrence and development of acute lung injury were searched and matched by the TCMSP database. The pharmacologic data and analysis platform of TCM were used to retrieve and screen for the volatile active components and the possible therapeutic targets of *Scutellaria baicalensis* georgi. In addition, acute lung injury was searched in the disease target database to identify the corresponding disease target proteins, thereby establishing a protein–protein interaction network. Finally, the effects of wogonin on the apoptotic and inflammatory factors in the acute lung injury cell model were analyzed experimentally.

**Results:** We identified 100 candidate targets and successfully constructed a complex target network. The targets identified by the above gene enrichment analysis played important roles in the autoimmune disease cell cycle apoptosis and related signaling pathways. The KEGG pathway analysis showed that most of the target genes were involved in the inflammatory response regulation of the TRP, PI3K-Akt, and IL-17 signaling pathways. The participation of wogonin in the specific regulatory pathways of PI3K-Akt signaling and IL-17 signaling was verified through experiments. In the lung-injured cell model, the results showed that wogonin inhibited the apoptosis of injured lung cells by inhibiting the expression of *BAD* gene and the activation of cleaved *caspase-3* gene while increasing Bcl-2 expression. In addition, wogonin inhibited the expression of the abovementioned inflammatory factors and further inhibited the inflammatory response in the lung injury cells.

**Conclusion:** The results of pharmacological network analysis can predict and explain the regulation mechanism of multi-target and multi-pathway of TCM components. This study identified the potential target and important pathway of wogonin in regulating acute lung injury. At the same time, the accuracy of network pharmacological prediction is also preliminarily verified by molecular biology experiment.

## Introduction

The pathogenesis of acute lung injury (ALI) essentially includes uncontrolled inflammatory response in the body, with injury of alveolar epithelial and pulmonary capillary endothelial cells as the main pathologic manifestations (Gouda and Bhandary, 2019; Mokra et al., 2019). The most important treatment strategy for ALI is mechanical ventilation. Drug therapy mainly comprises statins which have rich pharmacologic activities, can inhibit the release of inflammatory mediators and platelet aggregation, act as anticoagulant and antioxidant, and can improve endothelial function (Xu et al., 2019). In addition to statins, keratinized cell growth factor and neutrophil elastase inhibitors may be given for ALI (Hiroshima et al., 2017; Patel et al., 2018). In China, traditional Chinese medicine (TCM) is mainly used to treat ALI by regulating inflammatory mediators. *Scutellaria baicalensis* georgi is one of the most widely studied TCMs that were shown to have significant effects in the treatment of ALI; its active ingredients include baicalin and flavonoids (Ding et al., 2016; Meng et al., 2019; Peng et al., 2019; Zhi et al., 2020). However, only few have studied the effects of the volatile components of *Scutellaria baicalensis* georgi on ALI.

Network pharmacology is based on large data on background fusion system biology, molecular biology, and pharmacology, and a variety of emerging macroscopic to microcosmic disciplines on network computer platform (Poornima et al., 2016; Jain et al., 2018). It screens and builds a multilevel network, using a variety of database platform and computer software and data visualization that are more directed to interpret the correlation of TCM compounds with disease (Li et al., 2017; Sidders et al., 2018). Based on these, the volatile components in *S. baicalensis* were extracted by GC–MS experiment. The active components related with the development and progression of ALI were searched and matched with the TCMSP database. Then, based on network pharmacology, the volatile components of Radix Scutellariae and the network relationship among the ALI-related targets were simulated to clarify the mechanisms in ALI. In addition, we carried out a large number of biological experiments to validate the volatile components of Radix Scutellariae and to investigate the mechanism of wogonin for the treatment of ALI.

## Materials and Methods

### Extraction of Volatile Components From *Scutellaria baicalensis* by GC–MS

a) Sample preparation: 30 g of *Scutellaria* was crushed and sifted before being divided into three equal parts of 10 g each. Ethanol, methanol, and benzenol were respectively extracted using hot water for 4 h. After passing through a 0.22-µm ultrafiltration membrane, the filtrate was concentrated to 5 ml using a rotating evaporator, and again filtered through a 0.22-µm filter membrane to obtain samples in an injection bottle. The sample was set aside for GC–MS analysis (Agilent).
b) Gas chromatography conditions: the elastic quartz capillary column (RTX-5MS; 30 m × 0.25 mm, 0.25 µm) was heated by the program. The column temperature was successively increased to 210°C for 10 min, 220°C for 1 min, and 280°C for 10 min and was kept for 1 min; the determination time was 20 min in total. The shunting ratio was 20:1. The inlet temperature was 280°C and the column front pressure was 50 kPa. The injection volume was 1 μl, the carrier gas was high purity nitrogen (99.999%) and the flow rate was 1.1 ml/min.
c) Mass spectrum conditions: the EI ion source temperature was 200°C. The electron energy was 70 eV. The connector temperature was 250°C. The solvent delay was 3.5 min. The scanning range was 50–600 amu. The voltage of the electron multiplier was 1200 V.
d) Screening with the prior data: the corresponding compound ion flow chart was generated, according to the mass spectrum of each chromatographic peak fragments figure, literature report, and database data. The mass spectrum data was checked based on the peak mass-to-charge ratio and the relative abundance conditions, such as intuitive comparison. The chemical composition of the physical and chemical properties, as well as the pharmacologic action of the selected crude drug was determined.

### Screening of Active Ingredients in TCM

The bioactive compounds of TCM were downloaded from the TCMSP database. According to the absorption, distribution, metabolism, and excretion model of the body, oral bioavailability (OB) and drug similarity (DL) were used to screen the candidate active ingredients. DL ≥ 0.18 and OB ≥ 30% were set as the thresholds for screening the active ingredients. Thereafter, the chemical structure formula of each active ingredient compound was downloaded to predict the target gene.

### Prediction of the Action Targets of the Active Ingredients Contained in TCM

The compound structure of the active ingredient downloaded from the TCMSP database was uploaded to Baton-TCM and the SwissTargetPrediction database and its parameters were set as default values.

### Screening of Target Genes Associated With ALI

The target information related with ALI was obtained from the OMIM, NCBI-gene, and GeneCards database. By entering the information of the relevant gene of ALI, the target information related with ALI was finally obtained.

### Network Construction and Annotation Analysis Based on the Active Components of TCM and Targets of ALI

Cytoscape 3.6.1 software was used to establish the protein interaction network between the active components of TCM and the targets related with ALI, to integrate and extract the intersection network, and to perform topological structure analysis. The degree of topology eigenvalue in the network nodes was used as the index to screen the key targets. R language was used to extract the target gene information related with ALI. GO enrichment and KEGG pathway analyses were performed.

### Experimental Verification

#### Cell Culture and Treatments

BEAS-2B cells were cultured in an incubator with 37.5% carbon dioxide. According to previous report (Wang and Cui, 2019), wogonin concentration gradients 0,5,10, 15, 20, 25, 30, and 50 μmol/l, CCK-8 were designed to detect the effect of wogonin on cell activity. The final choice of wogonin was 50 μmol/l. The cells were pretreated with 50 μmol/l of wogonin for 8 h, followed by treatment with 50 g/ml of LPS for 16 h. CCK-8 detection was performed after reaching the time point.

#### Cell Activity Detection

The cells of each group at a specific time were placed into a 100-μl normal medium for each well, mixed with 10 μl of CCK-8, and incubated at 37°C for 1 h. The OD value at 450 nm was measured on a microplate analyzer for data analysis.

#### Western Blot

The cells were lyzed, the cellular proteins were extracted, and the protein concentration of the samples was quantified by an assay kit (Wanleibio Co., Ltd. Shenyang, China). Proteins were separated from each group of lysates (20 μg) using polyacrylamide gel and were transferred to polyvinylidene difluoride membranes (Millipore, Bedford, MA, United States). The membranes were washed in Tris-buffered saline with 0.5% Tween 20 (TBST) for 5 min and blocked in 5% skim milk for 1 h. The membranes were incubated with antibodies against caspase-3/cleaved caspase-3, BAD, and Bcl-2 (Wanleibio Co., Ltd. Shenyang, China, 1:2000 dilution) overnight at 4°C. Successively thereafter, the membranes were washed three times with TBST for 5 min each, incubated for 1 h at 37°C with rabbit HRP–conjugated anti-mouse antibody (1:1000) (Wanleibio Co., Ltd. Shenyang, China), and washed three times with TBST for 5 min each. We detected protein bands using an electrochemiluminescence agent (Wanleibio Co., Ltd. Shenyang, China). *β*-actin was used to normalize the band densities.

#### ELISA

Biochemical estimations of TNF-α, IL-1β, and IL-6 were performed using ELISA kits (Wanleibio Co., Ltd.), according to the manufacturer’s instructions.

### Statistical Analyses

All experimental data were tested for homogeneity and normality and were expressed as mean ± S.D. Analyses were performed using SPSS23.0 software. Comparison of the same time points was performed by independent sample t-test or analysis of variance. One-way analysis of variance was used and the data between the two groups were compared using the LSD test. *p* < 0.05 denoted statistical significance.

## Results

### Extraction of Volatile Components From *S. baicalensis* by GC–MS

**FIGURE 1:**
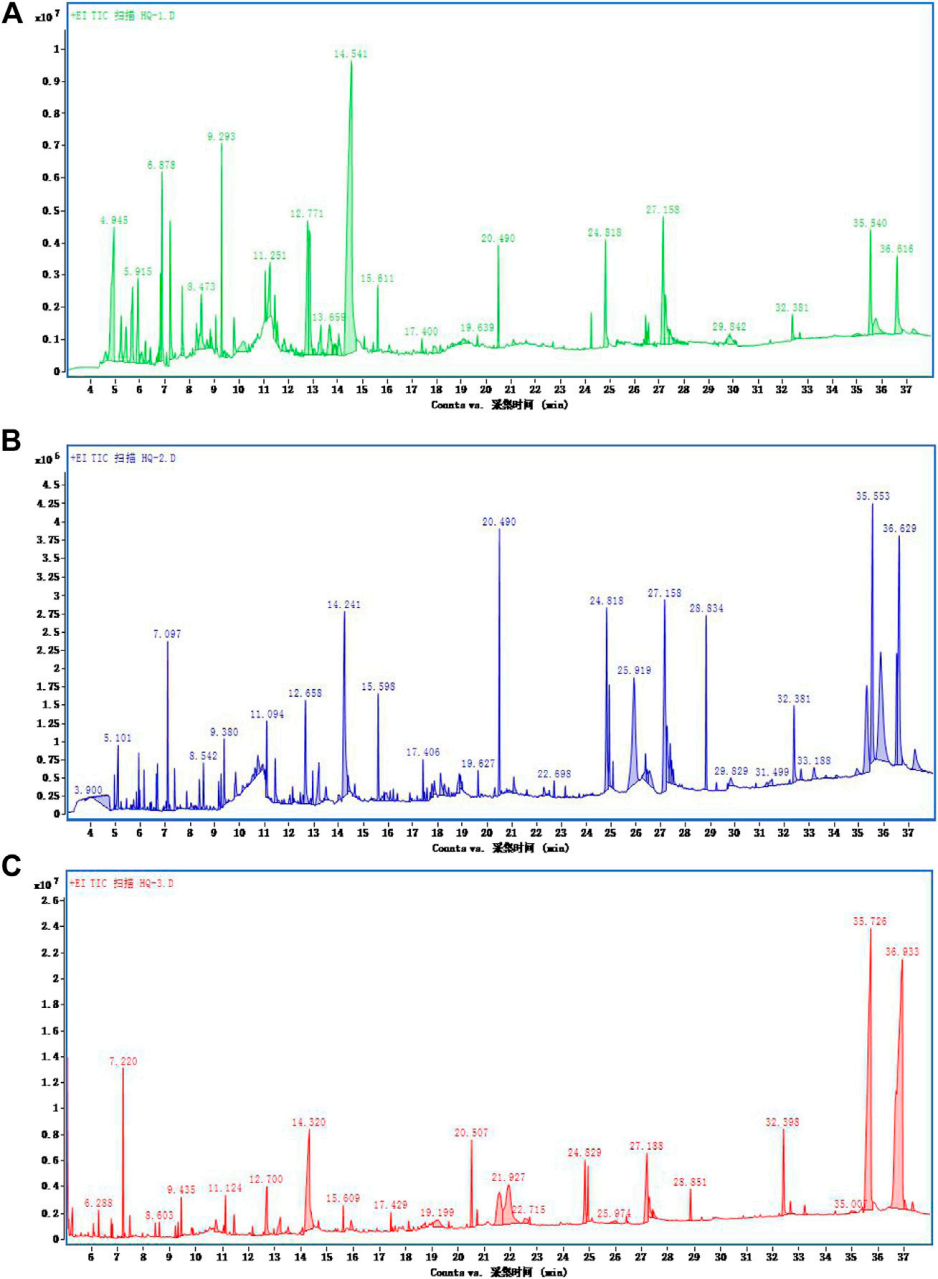
Ion flow diagram of *Scutellaria baicalensis georgi* detected by GC–MS. GC–MS detection ion flow diagram for extraction of *S. baicalensis* by **(A)** ethanol, **(B)** methanol, and **(C)** benzenol.

The volatile active ingredients of *S. baicalensis* were fully extracted by GC–MS using methanol, ethanol, and benzenol, and the results of the ion flow diagram are shown in Figure 1. Thereafter, the substances related with the development of ALI were searched and matched by TCMSP database. Finally, the volatile active ingredients of Scutellaria wogonin were identified by GC–MS analysis.

The extract of *Scutellaria baicalensis* was analyzed by GC–MS, and the volatile components of *Scutellaria baicalensis* were obtained by chromatography-mass spectrometry. Subsequently, the TCMSP database was used to identify the volatile components. As shown in Figure 1 and Table 1, a total of 31 volatile substances were obtained. Meanwhile, taking the peak area and peak area percentage as parameters and combining with 31 active components, the ratio of wogonin in the total 31 active volatile components was 49.38%. The phenylethanol extraction method had the highest volatile component content, which was 3.67 times and 3.95 times higher than that of ethanol extraction and methanol extraction, respectively. In the phenylethanol extraction method, the total amount of 43 compounds of wogonin accounted for 53.91%, the volatile components of wogonin were finally obtained.

**TABLE 1:** Volatile components of *Scutellaria baicalensis*.

Molecule name	CAS	OB (%)	DL
cis-5,8,11,14,17-Eicosapentaenoic acid	10417-94-4	45.66	0.21
1-Heptatriacotanol	105794-58-9	9.83	0.39
Hexadecanoic acid, methyl ester	112-39-0	18.09	0.12
delta-tocopherol	119-13-1	16.36	0.48
3-tert-butyl-4-hydroxyanisole	121-00-6	66.46	0.05
Ginsenoyne E	126146-63-2	36.53	0.13
Tributyl phosphate	126-73-8	27.76	0.06
1-Penten-3-one	1629-58-9	69.46	0
Dihydrooroxylin A	18956-18-8	38.72	0.23
Lactose	63-42-3	1.43	0.2
4-Mercaptophenol	637-89-8	60.34	0.01
5-Hydroxymethylfurfural	67-47-0	45.07	0.02
Panaxydol	72800-72-7	61.67	0.13
Cedrol	77-53-2	16.23	0.12
2-Methoxy-4-vinylphenol	7786-61-0	38.39	0.03
beta-Guaiene	88-84-6	19.91	0.07
4-Cyclopentene-1,3-dione	930-60-9	49.25	0.01
Benzoic acid, ethyl ester	93-89-0	27.58	0.03
Acetophenone	98-86-2	48.19	0.02
4H-Pyran-4-one, 2,3-dihydro-3,5-dihydroxy-6-methyl-	28564-83-2	37.8	0.03
1H-3a,7-Methanoazulene, 2,3,4,7,8,8a-hexahydro-3,6,8,8-tetramethyl-, [3 R-(3.alpha., 3a.beta., 7.beta., 8a.alpha.)]-	469-61-4	55.56	0.1
3-Furaldehyde	498-60-2	50.96	0.01
Orcinol	504-15-4	48.14	0.02
Di-epi-.alpha.-cedrene	50894-66-1	52.87	0.1
(1S,2S)-(+)-N-methylpseudoephedrine	51018-28-1	37.12	0.04
n-Hexadecanoic acid	57-10-3	19.3	0.1
2,5-Octadecadiynoic acid, methyl ester	57156-91-9	6.88	0.17
9,12-Octadecadienoic acid (Z,Z)-	60-33-3	41.9	0.14
2-Furancarboxaldehyde, 5-methyl-	620-02-0	43.92	0.01
Hexadecanoic acid, ethyl ester	628-97-7	18.99	0.14
Wogonin	632-85-9	30.68	0.23

### Identification and Analysis of the Effects of the Volatile Components of Scutellaria on Human Targets

Using the Baton-TCM and SwissTargetPrediction database, which predicted the corresponding human target information of the compound, we obtained 117 targets of wogonin in the human body.

### Identification and Analysis of Targets Related With ALI

**FIGURE 2:**
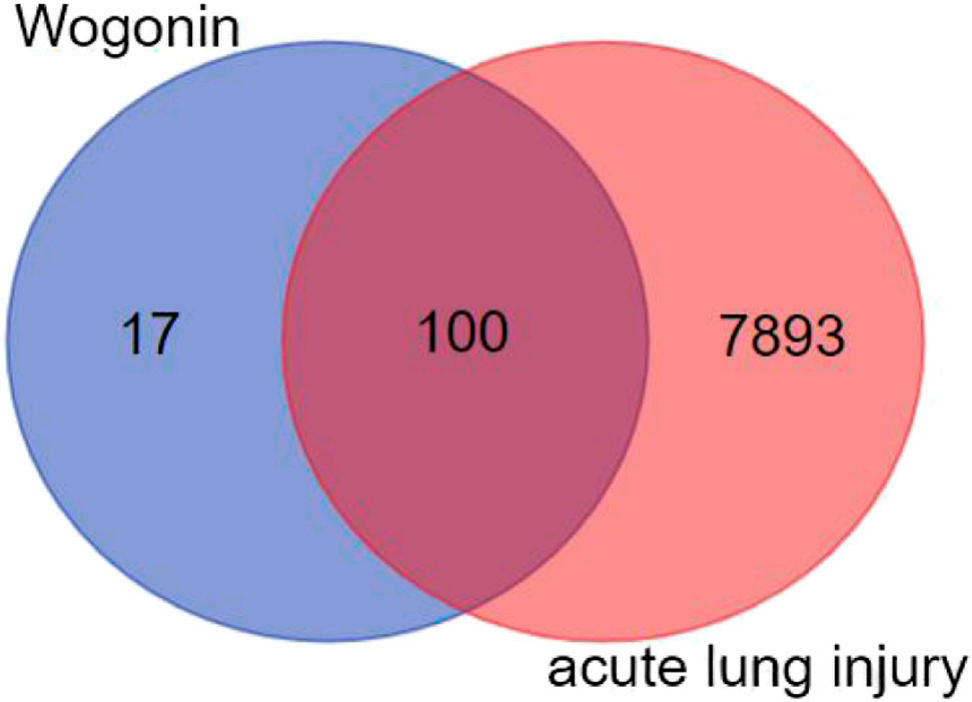
Venn diagram of the volatile components of *Scutellaria* and the targets related with ALI.

**FIGURE 3:**
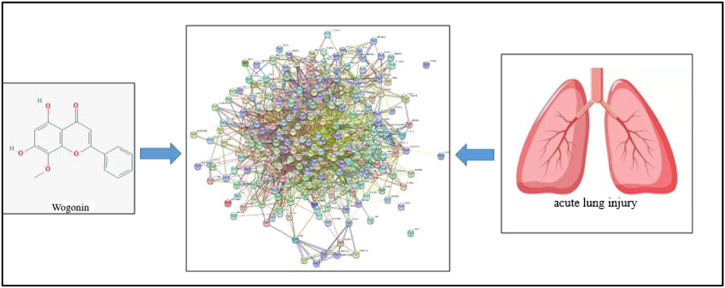
Regulatory network model of the volatile components of *Scutellaria* acting on ALI.

We retrieved a list of genes associated with ALI: 106 genes were obtained from OMIM, 7966 genes were obtained from the GENECARDS tool, and 1265 genes were obtained from NCBI-GENE. Combination of the three databases obtained 7993 gene information related with ALI. After statistical analysis of the targets of the Scutellaria volatile components and the related targets of ALI, a combination of 100 targets were obtained (Figure 2). Using Cytoscape software, the network of the related targets of Scutellaria volatile components acting on ALI was analyzed to obtain the protein interaction network (Figure 3).

### Functional Enrichment Analysis of TCM Targets

**FIGURE 4:**
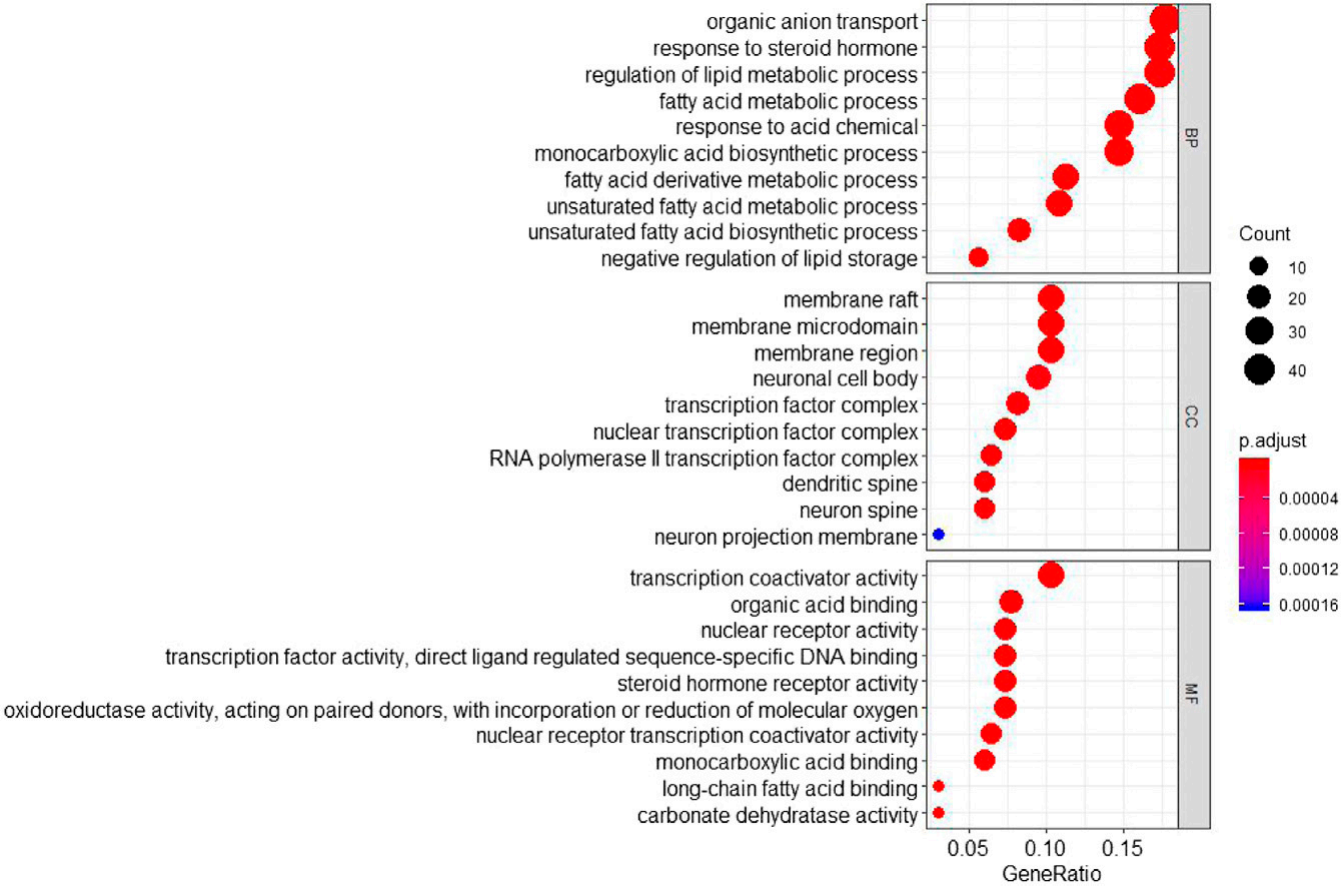
GO annotation of *Scutellaria* volatile components acting on the target of ALI.

**FIGURE 5:**
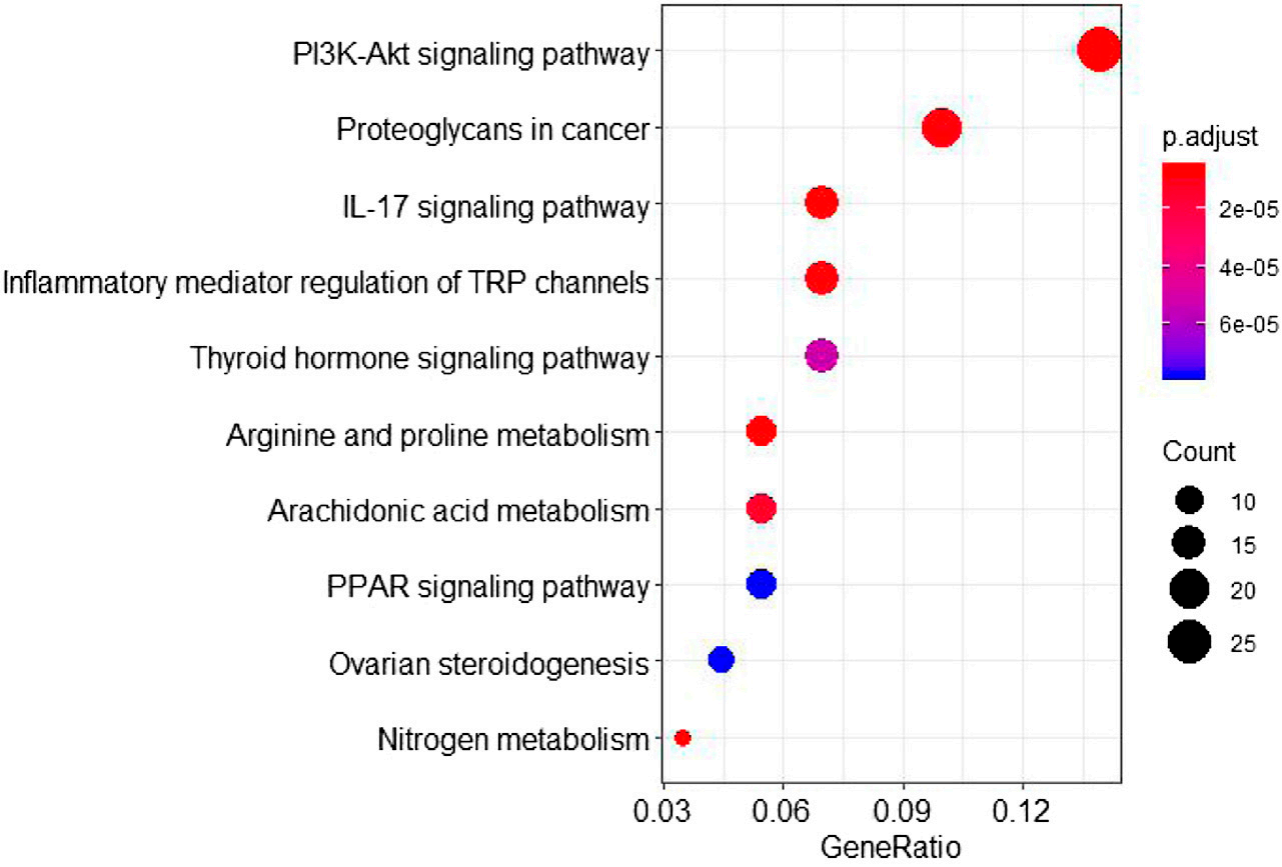
KEGG pathway analysis of the *Scutellaria* volatile components acting on the targets of ALI.

**FIGURE 6:**
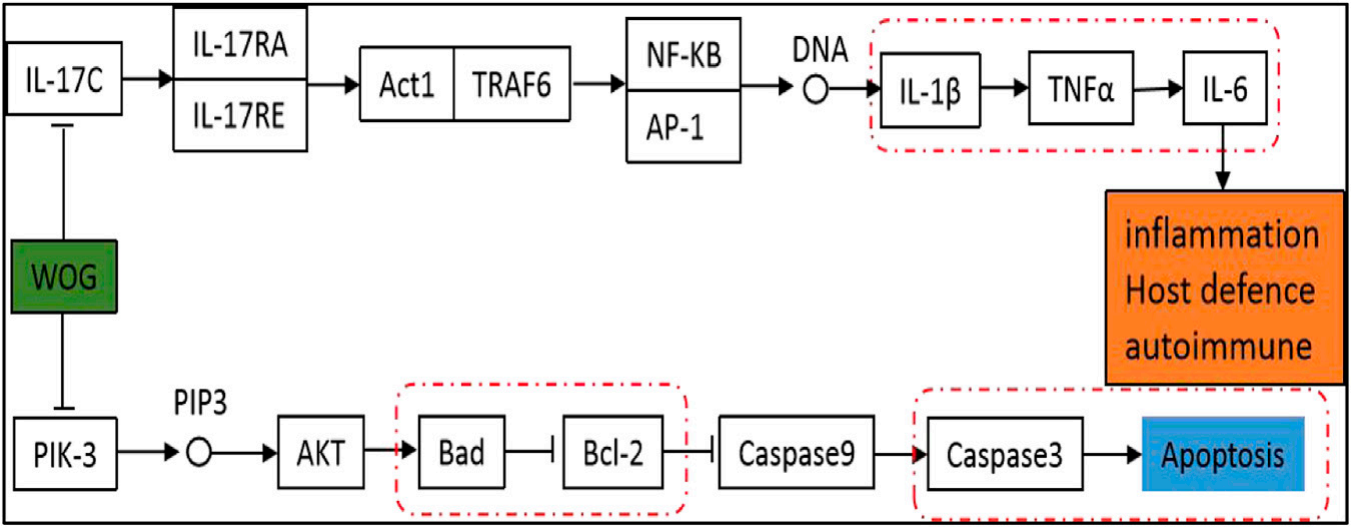
Pathway diagram of the wogonin involved in the PI3K-Akt and IL-17 signaling pathways.

The GO annotation analysis showed that the target of the Scutellaria volatile components on ALI was related with the regulation of fatty acid metabolism (Figure 4). The KEGG pathway analysis showed that most of the target genes were involved in the inflammatory response regulation of the TRP, PI3K-Akt, and IL-17 signaling pathways (Figure 5). ALI was mainly induced by LPS, resulting in a severe inflammatory response in the body. Our analysis showed relationship with the apoptotic signaling pathway of PI3K-Akt and the inflammatory factor signaling pathway of IL-17. According to KEGG results, the specific regulation paths of the two signaling pathways were further obtained and the results are shown in Figure 6.

Based on the results of the network pharmacology analysis, we used biological experiments to verify the molecular model of the Scutellaria volatile components acting on ALI.

### Effects of Wogonin on the Key Targets in the Lung Injury Cell Model

**FIGURE 7:**
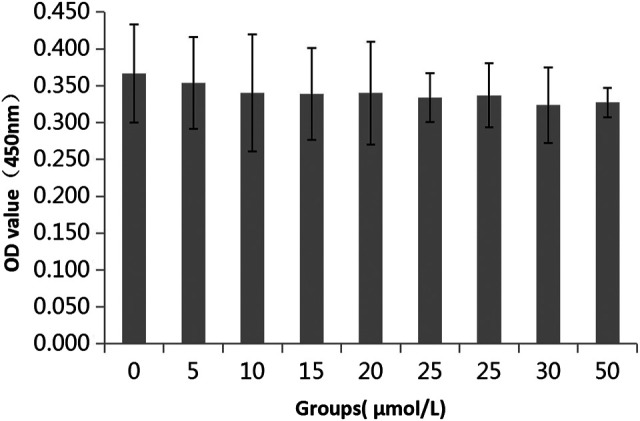
Effect of wogonin on BEAS-2B cell viability.

The effect of wogonin on the activity of BEAS-2B cells was not significantly different under different concentration gradients, and the effect of wogonin on cell proliferation was not significantly different between all concentration gradients and without the addition of wogonin. In conclusion, different concentrations of wogonin (5, 10, 15, 20, 25, 30, and 50 μmol/l) had no effect on the activity of BEAS-2B cells (Figure 7). Finally, in order to increase the effect of wogonin on lung injury cells and facilitate the subsequent study of the mechanism, we chose the maximum concentration of 50 μmol/l in the concentration gradient for subsequent experimental studies.

**FIGURE 8:**
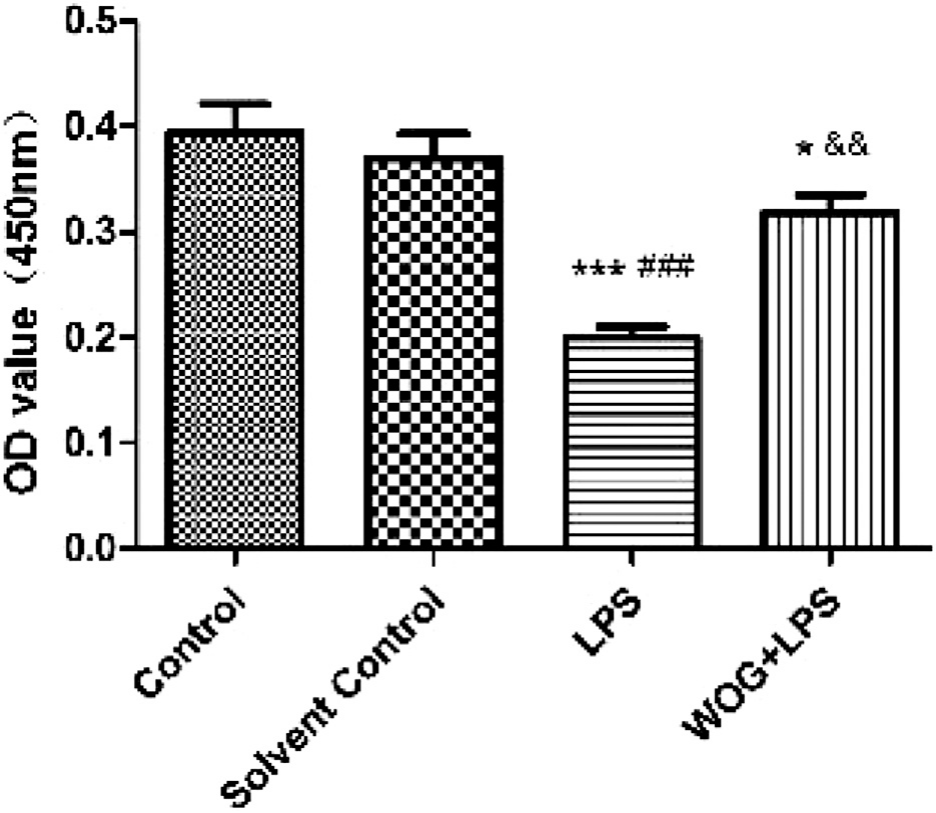
Effect of wogonin on the activity of BEAS-2B cells with LPS. ^*^ *p* < 0.05, ^**^ *p* < 0.01,^***^ *p* < 0.001, compared with the control group. ^#^ *p* < 0.05, ^##^ *p* < 0.01, ^###^ *p* < 0.001, compared with the solvent control group. ^&^ *p* < 0.05, ^&&^ *p* < 0.01, ^&&&^ *p* < 0.001, compared with the LPS group.

For the subsequent tests, the concentration of wogonin was selected as 50 μmol/l, as previously reported (Wang and Cui, 2019). In the lung injury cell model, the cell activity of the normal BEAS-2B cells decreased significantly after the addition of LPS; the model was successfully constructed. After the addition of wogonin, the activity of the BEAS-2B cells significantly increased (Figure 8).

**FIGURE 9:**
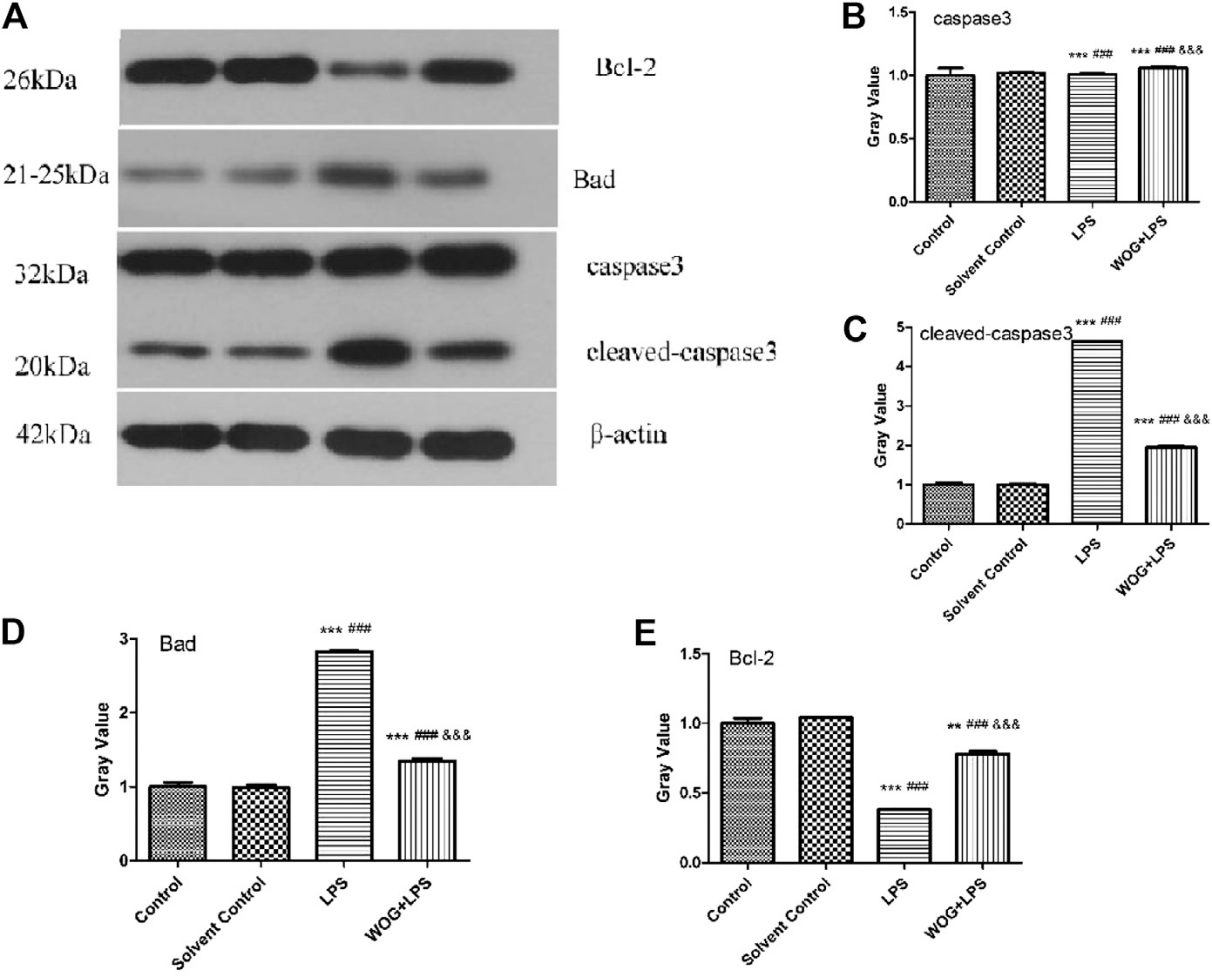
Effect of wogonin on the BEAS-2B cells with LPS **(A)** Western blot analysis and **(B–E)** Gray analysis inhibited expressions of caspase-3/cleaved caspase-3, BAD, and Bcl-2 proteins. ^*^ *p* < 0.05, ^**^ *p* < 0.01,^***^ *p* < 0.001, compared with the control group. ^#^ *p* < 0.05, ^##^ *p* < 0.01, ^###^ *p* < 0.001, compared with the solvent control group. ^&^ *p* < 0.05, ^&&^ *p* < 0.01, ^&&&^ *p* < 0.001, compared with the LPS group.

In order to further verify the antiapoptotic mechanism of wogonin in lung injury, we analyzed the regulatory effect of wogonin on the PI3K-Akt apoptotic signaling pathway, according to the amplification analysis results of KEGG. On the Western blot analysis, we detected the expression of the apoptotic regulation genes, such as *caspase-3/cleaved caspase-3*, *BAD*, and *Bcl-2*. The results showed that compared with the blank control group, the LPS group had significantly increased expressions of *BAD* and *cleaved caspase-3*, significantly decreased the expression of *Bcl-2*, and no significant change in the expression of *caspase-3*. Compared with the LPS group, the LPS + wogonin group had significantly decreased expressions of *BAD* and *cleaved caspase-3*, significantly increased expression of *Bcl-2*, and no significant change in the expression of *caspase-3* (Figure 9).

**FIGURE 10:**
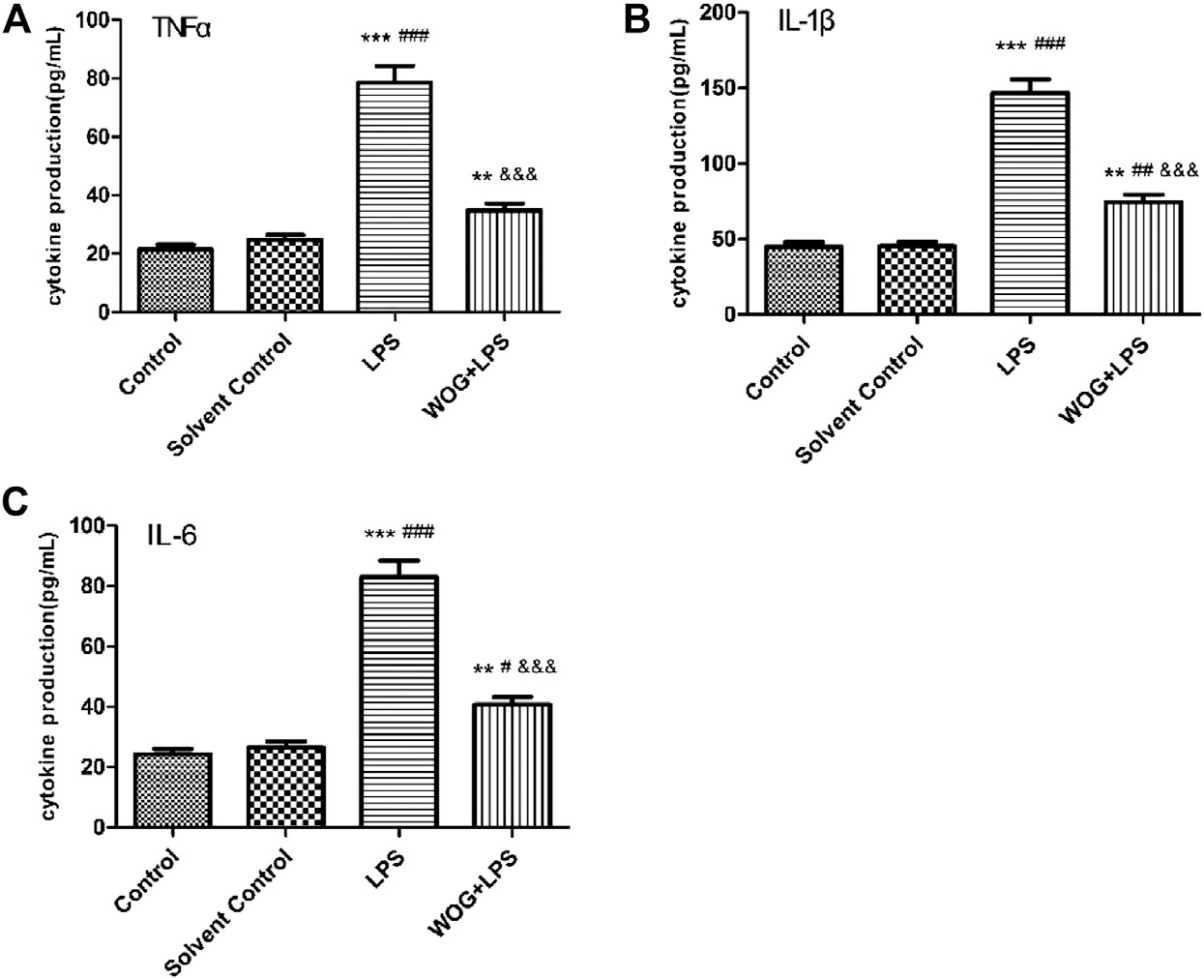
Effect of wogonin on the BEAS-2B cells with LPS. The expressions of **(A)** TNF-α, **(B)** IL-1β, and **(C)** IL-6 are inhibited. ^*^ *p* < 0.05, ^**^ *p* < 0.01,^***^ *p* < 0.001, compared with the control group. ^#^ *p* < 0.05, ^##^ *p* < 0.01, ^###^ *p* < 0.001, compared with the solvent control group. ^&^ *p* < 0.05, ^&&^ *p* < 0.01, ^&&&^ *p* < 0.001, compared with the LPS group.

In order to further verify the anti-inflammatory mechanism of wogonin in ALI, we analyzed the regulatory effect of wogonin on the IL-17 signaling pathway, according to the amplified analysis results of KEGG. TNF-α, IL-1, and IL-6 secretions were significantly increased in the LPS group, compared with those in the blank control group. Compared with the LPS group, the LPS + wogonin groups had significantly decreased secretion of inflammatory factors. As shown in Figure 10 and Table 2, wogonin inhibited the expression of the inflammatory factors in the lung injury cell model.

**TABLE 2:** Effects of wogonin on the BEAS-2B cells with LPS on the expressions of TNF-α, IL-1β, and IL-6.

Group	TNF-α	IL-1β	IL-6
Control	21.577 ± 2.712	44.776 ± 5.462	24.258 ± 2.936
Solvent control	24.738 ± 2.988	45.219 ± 4.889	26.484 ± 3.374
LPS	78.438 ± 10.109	146.657 ± 15.757	82.992 ± 9.485
LPS + wogonin	34.896 ± 3.910	74.352 ± 8.463	40.716 ± 4.324

## Discussion

At present, most conventional TCM use water-boiled processing to extract the hydrothermally soluble material components. In recent years, the pharmacologic effects of nonhydrothermally soluble substances contained in Chinese medicines have gradually gained the attention of researchers (Zorzetto et al., 2015). However, because of the pharmacologic effects of the volatile components in TCM, research and development of these volatile components have been limited by the prolonged period of animal experiments. Therefore, only few have studied the pharmacologic effects of the volatile oils in TCM. With the discovery of modern pharmacologic studies in recent years, more studies on the chemical properties and pharmacologic activities of the volatile substances in Chinese herbal medicines have been conducted. The volatile components of Chinese herbal medicines not only have analgesic, antibacterial, and anti-inflammatory effects to improve myocardial function but also have curative effects to prevent hypertension and hyperlipidemia (Singh et al., 2015; Wang et al., 2016; Zhou et al., 2016; Bui et al., 2017). In this study, the volatile components of *Scutellaria baicalensis* georgi were extracted by GC–MS, and the active components related with the development and progression of ALI were matched with the active components of Scutellaria baicalin in combination with the TCMSP database. The active volatile components of Scutellaria (wogonin) were screened for their effects on lung injury, the network analysis on the relevant targets of wogonin acting on ALI were conducted, and the protein interaction network was obtained. Further KEGG and GO analyses found that the role of wogonin was mainly concentrated in the apoptotic signaling pathway of PI3K-Akt and the inflammatory factor signaling pathway of IL-17. Therefore, we hypothesized that wogonin may exert therapeutic effects on ALI through negative regulation of apoptotic factors and pro-inflammatory factors. Furthermore, we established a lung injury cell model *in vitro* using LPS-induced BEAS-2B cells, verified the systematic pharmacologic prediction results, and discussed the mechanism of wogonin in treating ALI.

In the PI3K/AKT signaling pathway, *Bcl-2* is a gene that inhibits the apoptosis caused by a variety of cytotoxic factors and enhances the resistance of cells to most factors that cause DNA damage (Ashkenazi et al., 2017; Zhang et al., 2020). The downstream factor *BAD* is a target proapoptotic gene that regulates cell apoptosis and survival. In some studies on ALI induced by OA experiments, detecting the expression of VEGF, Bcl-2, BAD, and PI3K/AKT in serum of pulmonary bronchial lavage, the results showed that the increased Bcl-2 protein expression was associated with the activation of AKT, the activation of AKT inhibited the expression of BAD factors, thereby, inhibiting lung cell apoptosis; this effect was achieved by the PI3K/AKT pathway. Caspase is the executor of apoptosis and other families regulate apoptosis by directly or indirectly inhibiting the activation of the members of the Caspase family (Bolívar et al., 2019). These proteases exist in the normal state as inactive progenases and are activated by apoptotic signals that stimulate the cleavage of their specific aspartic acid residues (Larsen and Sørensen, 2017). Caspase in the upstream can sequentially activate caspase downstream, forming a cascade reaction and transmitting apoptotic signals to apoptotic substrates. Both extracellular and intracellular apoptosis signals need to activate caspase-6 and caspase-7 through caspase-3 and activate various enzymes to directly lead to the characteristic apoptotic manifestations, such as DNA degradation cell wrinkling (Sergio et al., 2018). Therefore, caspase-3 is the central link in the apoptotic signal transduction process and the detection of activated caspase-3 level can reflect the apoptotic process (Messer et al., 2013). In our experiment, the cell expressions of Bcl-2, BAD, caspase-3, and cleaved caspase-3 were detected by Western blot. The results showed that, in the lung injury cell model, wogonin inhibited the expression of the *BAD* gene and activation of the *cleaved caspase-3* gene and increased the expression of *Bcl-2*, thereby, inhibiting the apoptosis of lung injury cells. In summary, these data indicated that wogonin effectively inhibited the apoptosis of lung injury cells and confirmed the results of functional enrichment analysis.

Multiple inflammatory cytokines are expressed in the lung tissue cells in ALI, and the IL-17 signaling pathway was involved in the secretion of TNF-α, IL-1β, and IL-6 (Ding et al., 2017). TNF-α is an important bioactive medium in the human body and can activate TNF-α to induce cell apoptosis and IL-6 expression and promote fibroblast proliferation (Lai et al., 2019). Il-1β, which is an endogenous source of heat, is a proinflammatory cytokine that triggers immune and inflammatory responses and can be synthesized and secreted by a variety of cells. Studies have shown that IL-1β was a promoter of inflammatory amplification and was involved in the development of inflammation (Jia et al., 2019). In this experiment, LPS was used to construct a lung injury cell model, and TNF-α, IL-6, and IL-1β were detected by ELISA. The results showed that wogonin inhibited the expression of TNF-α, IL-6, and IL-1β in lung injury cells. Therefore, these data indicated that wogonin effectively inhibited the inflammatory response of lung injury and confirmed the results of functional enrichment analysis.

In summary, this study preliminarily identified the key targets and important pathways of wogonin regulating acute lung injury through network pharmacology. At the same time, we carried out molecular biological verification on the key targets, and the experimental results also confirmed the accuracy of network pharmacological prediction. Using network pharmacological methods, this study provides an alternative strategy to help us understand the specific mechanism of traditional Chinese medicines such as wogonin in the treatment of diseases such as acute lung injury. At the same time, we will design more reasonable experiments to carry out in-depth mechanism research in the future.

## Data Availability

The original contributions presented in the study are included in the article/Supplementary Material; further inquiries can be directed to the corresponding authors.
